# Enhanced specific antitumor immunity of dendritic cells transduced with the glypican 3 gene and co-cultured with cytokine-induced killer cells against hepatocellular carcinoma cells

**DOI:** 10.3892/mmr.2015.3239

**Published:** 2015-01-22

**Authors:** YULIANG WANG, YINLONG WANG, HONG MU, TAO LIU, XIAOBO CHEN, ZHONGYANG SHEN

**Affiliations:** 1 Department of Clinical Laboratory Medicine, Tianjin First Central Hospital, Key Laboratory for Critical Care Medicine of the Ministry of Health, Tianjin 300192, P.R. China; 2 Department of Transplantation Surgery, Tianjin First Central Hospital, Key Laboratory for Critical Care Medicine of the Ministry of Health, Tianjin 300192, P.R. China; 3 Department of Hernia and Abdominal Wall Surgery, Union Medicine Center, Tianjin 300121, P.R. China; 4 Union Stem and Gene Engineering Co., Tianjin 300384, P.R. China

**Keywords:** dendritic cells, cytokine-induced killer cells, hepatocellular carcinoma, glypican 3, cytotoxicity

## Abstract

Dendritic cell (DC)-based cancer immunotherapy requires an immunogenic tumor-associated antigen and an effective therapeutic strategy. Glypican 3 (GPC3) is a valuable diagnostic marker and a potential therapeutic target in hepatocellular carcinoma (HCC). The present study investigated whether DCs transduced with the GPC3 gene (DCs-GPC3) and co-cultured with autologous cytokine-induced killer cells (CIKs) may induce a marked specific immune response against GPC3-expressing HCC cells *in vitro* and *in vivo*. Human DCs were transfected with a green fluorescent protein plasmid with GPC3 by nucleofection and then co-cultured with autologous CIKs. Flow cytometry was used to measure the phenotypes of DCs and CIKs. The co-cultured cells were harvested and incubated with HCC cells and the cytotoxicity of the CIKs was assessed by nonradioactive cytotoxicity assay. The anti-tumor activity of these effector cells was further evaluated using a nude mouse tumor model. The results demonstrated that DCs-GPC3 significantly promoted the autologous CIKs differentiation, as well as anti-tumor cytokine interferon-γ secretion. In addition, DCs-GPC3-CIKs significantly enhanced the cytotoxic activity against GPC3-expressing HepG2 cells, indicating a GPC3-specific marked immune response against HCC cells. The *in vivo* data indicated that DCs-GPC3-CIKs exhibited significant HepG2 cell-induced tumor growth inhibition in nude mice. The results of the present study provided a new insight into the design of personalizing adoptive immunotherapy for GPC3-expressing HCC cells.

## Introduction

Liver cancer, the most common form of which is hepatocellular carcinoma (HCC), is the fifth most common malignant tumor and the third leading cause of cancer-associated mortality worldwide. Each year ~500,000 new cases of HCC are diagnosed worldwide (1,2). Surgical and nonsurgical therapeutic treatments include tumor resection, liver transplantation, radiofrequency (thermal) ablation, percutaneous ethanol injection and transarterial chemoembolization (3). Despite these methods, the prognosis for HCC patients remains poor and tumor recurrence rates remain high (4). Therefore, novel therapeutic approaches are required to improve the treatment outcome for patients with HCC (5).

Tumor adoptive immunotherapy has been demonstrated to have potential as an adjunct treatment to control the disease. This approach can be efficiently employed for the eradication of residual cancer cells and prevention or delay of tumor relapse (NEW 1 – 6). With the development of tumor adoptive immunology, progress has been achieved in preclinical studies and clinical practice (7). Dendritic cells (DCs) and cytokine-induced killer cells (CIKs) have been demonstrated to possess high *in vitro* and *in vivo* antitumor and cytotoxic activities against HCC cells (8–10). DCs are the most potent antigen-capturing and antigen-presenting cells, with the ability to capture, process and present tumor antigens to naïve cells, and stimulate a marked immune response against these antigens. The antigen-presenting ability of DCs makes them attractive vehicles for the delivery of therapeutic tumor vaccines and provides a suitable platform for vaccine development (11). In 2010, the first DC-associated cancer vaccine for prostate cancer therapy received approval from the U.S. Food and Drug Administration (12). CIKs are obtained from human peripheral blood mononuclear cells stimulated by interferon (IFN)-γ, interleukin (IL)-2 and cluster of differentiation (CD)3 monoclonal antibodies. CIKs can express the surface markers of T cells and natural killer (NK) cells (13). The characteristic CD3^+^CD56^+^ CIKs phenotype has been demonstrated to exhibit a major histocompatibility complex (MHC)-unrestricted tumor killing ability, *in vitro* and in medical practice (14). The CIKs that possess the ability to attack tumor cells are expressed on the cell surface of CD3/CD56. In addition, CIKs have superior antitumor activity against a variety of cancer types, evident by their co-culturing with antigen-loaded DCs. Therefore, as a nontoxic, efficient and adoptive immunotherapeutic strategy, the use of a vaccine of DCs co-cultured with CIKs may increase the potential of specific immune response against HCC.

Studies performed by the authors of the present study and by other researchers have investigated the expression, function and regulation of carcinoembryonic antigen glypican 3 (GPC3) which has been found to be overexpressed in HCC tissues and may serve as a potential diagnostic biomarker and therapeutic target for this disease (15–17). GPC3, a 70 kDa protein of 580 amino acids, is a heparan sulfate proteoglycan that is positioned on the cell surface using a mechanism involving a glycosylphosphatidylinositol anchor. In addition, GPC3 promotes the growth of HCC cells through the stimulation of the canonical Wnt signaling pathway (18). In HCC tumors, GPC3 is overexpressed and correlates with poor prognosis, as well as functioning as a secretory protein released from the cell membrane surface to the extracellular environment (19). Therefore, GPC3 may serve as a tumor-associated antigen (TAA) target for immunotherapy against HCC. Considering the aforementioned properties, the present study analyzed the effectiveness of CIKs co-cultured with autologous GPC3-transduced DCs against GPC3-expressing HCC cells, *in vitro* and *in vivo*. The present study aims to provide new insight into the design of DC-based tumor vaccine strategies for personalizing adoptive immunotherapy.

## Materials and methods

### Animals and cell line

Nude mice (age, 6–8 weeks) were purchased from the Academy of Military Medical Science (Beijing, China). The mice were housed under specific pathogen-free conditions. All the experiments were performed according to the National Institutes of Health Guide for Care and Use of Laboratory Animals (National Institutes of Health, Bethesda, MD, USA) and were approved by the Bioethics Committee of Tianjin First Central Hospital (Tianjin, China). The human HCC cell line, HepG2 (GPC3-expressing cell line), was purchased from the American Type Culture Collection (Rockville, MD, USA) and maintained in the Key Laboratory for Critical Care Medicine of the Ministry of Health (Tianjin, China). The cells were cultured in complete RPMI 1640 medium [RPMI 1640 (Invitrogen Life Technologies, Carlsbad, CA, USA) supplemented with 10% heat-inactivated fetal bovine serum (Gibco Life Technologies, Carlsbad, CA, USA), 100 U/ml penicillin and 100 mg/ml streptomycin (Sigma-Aldrich, St. Louis, MO, USA)] at 37°C in a 5% CO_2_ atmosphere.

### Generation of DCs and CIKs

DCs and CIKs were generated from peripheral blood mononuclear cells (PBMCs) of consenting healthy volunteers according to our protocol approved by the ethics committee of Tianjin First Central Hospital. DCs and CIKs were generated as described previously (20). Briefly, PBMCs were isolated from whole blood by Ficoll density gradient centrifugation using a commercially lymphocyte separation medium (Sigma-Aldrich) and centrifuged at 400 × g for 25 min (NEW 2 – 21). Next, the cells were allowed to adhere in six-well plates (Corning Life Sciences Tewksbury, MA, USA) at a density of 5×10^6^ cells/ml for 2 h at 37°C in complete RPMI 1640 medium. The adherent and non-adherent cells were collected for generating DCs and CIKs, respectively. To generate DCs, the adherent cells were cultured in complete RPMI 1640 medium with 1,000 U/ml recombinant human (rh) granulocyte-macrophage colony-stimulating factor and 500 U/ml rhIL-4 (R&D Systems, Minneapolis, MN, USA) at 37°C in a humidified atmosphere of 5% CO_2_ and immature DCs were obtained. The medium along with the necessary cytokines were replaced every three days. On day 6, a further 1,000 U/ml tumor necrosis factor (TNF)-α was added to the DC sample to induce maturation. To generate CIKs, the non-adherent PBMCs were prepared and grown in complete RPMI 1640 medium with 1,000 U/ml rhIFN-γ. After 24-h incubation, 50 ng/ml mouse anti-human CD3 monoclonal antibody and 1,000 U/ml IL-2 were added. The CIKs were incubated at 37°C in a humidified atmosphere of 5% CO_2_ and subcultured every three days with cytokine replenishment.

### Transduction of DCs with the GPC3 gene

The recombinant plasmid green fluorescent protein (pGFP)-GPC3 eukaryotic expression vector was constructed and maintained at the Key Laboratory for Critical Care Medicine of the Ministry of Health (Tianjin, China). Briefly, the pDONR223-GPC3 plasmid (full length GPC3 cDNA) was ligated into a pcDNA-DEST53 vector containing GFP (Invitrogen Life Technologies) with recombinase. The recombinant pGFP-GPC3 was amplified in *E. coli* DH5α competent cells and isolated with Takara MiniBEST plasmid purification kit (Takara Bio, Inc., Otsu, Japan). The correct pGFP-GPC3 plasmid sequence was verified using DNA analysis. The DCs were transduced using the Amaxa^®^ Nucleofector^®^ apparatus (Lonza Cologne GmbH, Cologne, Germany), according to the manufacturer’s instructions. Briefly, on day 6, 5×10^6^ immature DCs were cultured in serum-free growth medium (Gibco Life Technologies) without antibiotics prior to nucleofection. The cells were gently resuspended in 100 μl human electroporation buffer (Lonza Cologne GmbH) at a concentration of 2×10^6^ cells/100 μl and then transferred to a sterile Amaxa^®^ nucleofection cuvette (Lonza Cologne GmbH). Subsequently, the immature DCs were incubated with 2 μg pEGFP-GPC3 or empty vector containing GFP. The cells were electroporated using of the appropriate nucleofection program (as recommended in the manufacturer’s instructions) and immediately transferred into six-well plates containing fresh pre-warmed culture medium at 37°C with the necessary cytokine (TNF-α) and serum. DCs were incubated at 37°C for 24 h to induce maturation and were termed as the DCs-GPC3 group. DCs transduced with pcDNA3 (DC-pcDNA3) were used as the control group. After 24 h of incubation, DCs-GPC3 viability was assessed using trypan blue exclusion (Sigma-Aldrich) and the transfection efficiency of the cells was assessed by the extent of GFP expression using Ni-U fluorescence microscopy (Nikon Corporation, Tokyo, Japan) and fluorescence-activated cell sorting (FACS) flow cytometric analysis was performed using a FACSCalibur flow cytometer (BD Biosciences, Franklin Lakes, NJ, USA). The DCs were then collected for subsequent experiments.

### GPC3 expression in DCs-GPC3

The expression of GPC3 in DCs-GPC3 was detected at the transcriptional and translational levels. Following transfection for 48 h, DCs-GPC3 were collected and the total RNA or total protein was prepared for detection by TaqMan reverse transcription-polymerase chain reaction (RT-PCR) or western blotting, respectively. Non-transduced mature DCs and DCs-pcDNA3 were evaluated in parallel as controls. Primer Premier V5.0 software was used to design the primers according to human gene sequences (GenBank database, www.ncbi.nlm.nih.gov/genbank). Primers were synthesized by Integrated DNA Technologies (Coralville, IA, USA). The PCR primers used for GPC3 were as follows: forward, 5′-AGAGGCCTTTGAAATTGT-3′, and reverse 5′-AAATACTTTCAGGTCACGTC-3′; and the probe 5′-FAM-ATGCCAAGAACTACACCAATGCTAMRA-3′ (22). The conditions for each PCR reaction were as follows: 15 min at 95°C, followed by 40 cycles of denaturation for 20 sec at 95°C and annealing/extension for 60 sec at 60°C. The level of expression was represented as 2^−ΔCt^, where ΔCt was calculated as: (copy number of target molecule)/(copy number of β-actin). For western blot analysis, the proteins were resolved on an SDS denaturing polyacrylamide gel and then transferred onto nitrocellulose membranes (EMD Millipore, Billerica, MA, USA). A primary rabbit anti-human polyclonal antibody to GPC3 (sc-11395; 1:500) or an endogenous control β-actin (sc-7210; 1:500) were incubated with the membranes first and then with horseradish peroxidase-conjugated goat anti-rabbit IgGFc secondary antibodies (sc-2004; 1:2,000) (All antibodies from Santa Cruz Biotechnology Inc., Santa Cruz, CA, USA). Protein expression was assessed by enhanced chemiluminescence (EMD Millipore), and the bands were captured using a FluorChem FC2 Imaging System (ProteinSimple, San Jose, CA, USA).

### Phenotypic characterization

To ensure that the DCs were mature, DCs-GPC3 and DCs were collected on day 8 and resuspended in cold FACS buffer. The cells were immunostained with fluorescein isothiocyanate (FITC)-labeled or phycoerythrin (PE)-labeled mouse monoclonal antibodies against human CD80, CD83, CD86, human leukocyte antigen (HLA)-DR or an isotype control. All the monoclonal antibodies used in this study were obtained from BD Pharmingen, San Diego, CA, USA. The cells were incubated with antibodies on ice for 30 min, washed twice with phosphate-buffered saline (PBS) and resuspended. Next, phenotypic characterization was performed by flow cytometric analysis using the FACSCalibur flow cytometer (BD Biosciences). CIKs were harvested on day 8 and co-cultured for a further seven days with autologous DCs, DCs-pcDNA3 or DCs-GPC3 at a ratio of 1:5 to produce DCs-CIKs, DCs-pcDNA3-CIKs or DCs-GPC3-CIKs, respectively. Subsequently, CIKs were harvested on day 14 and their cytotoxicity was assessed. Phenotypic characterization of CIKs was conducted with antibodies against CD3, CD8 and CD56 (BD Pharmingen). Next, the cells were incubated with the corresponding antibodies on ice for 15 min and then washed with PBS, and flow cytometric analysis was performed.

### Analysis of IFN-γ-secreting CIKs

IFN-γ-secreting cells were detected on day 7 of the co-culture using intracellular staining and flow cytometry (23). Briefly, the aforementioned proliferative CIKs, DCs-CIKs, DCs-pcDNA3-CIKs or DCs-GPC3-CIKs were suspended in complete RPMI 1640 and stimulated for 4 h with 25 ng/ml phorbol 12-myristate 13-acetate, 1 μM ionomycin and 2 μM monensin (Sigma-Aldrich). Following washing with PBS, the cells were stained with FITC-conjugated mouse anti-human CD3 monoclonal antibody (BD Pharmingen) for 30 min at 4°C, washed with PBS and then permeabilized with FACS permeabilizing solution (BD Pharmingen) for a further 10 min at room temperature. The samples were incubated with PE-labelled mouse anti-human INF-γ monoclonal antibody (BD Pharmingen) for 30 min at room temperature in the dark, washed with PBS and analyzed by flow cytometry.

### Cytotoxicity assay

A nonradioactive cytotoxicity assay kit (Promega Corp., Madison, WI, USA), lactate dehydrogenase (LDH) release, was used to measure the cytotoxic activity on target cells, according to the manufacturer’s instructions. Briefly, the target cells, GPC3-expressing HepG2, were plated in triplicate in 96-well culture plates and incubated with the various effector cells (CIKs, DCs-CIKs, DCs-pcDNA3-CIKs and DCs-GPC3-CIKs) with an effector to target (E/T) ratio of 20:1 or 50:1. Maximal release of LDH was performed by completely lysing target cells. Target cells without effector cells were used as negative controls (spontaneous release). Cytotoxicity was calculated as follows: percentage cytotoxicity (%) = [(experimental release - spontaneous release of effector cells - spontaneous release of target cells) / (maximal release of target cells - spontaneous release of target cells)] ×100.

### Animal testing

Tumors were generated by subcutaneous inoculation with 1×10^7^ HepG2 cells in 0.2 ml of PBS into the right flank of each nude mouse with a 100% incidence rate on day 7. The mice were randomly divided into four groups (n=4 each) and subjected to treatment. Subsequently, the four groups were injected locally with DCs-GPC3-CIKs (1×10^7^ cells), DCs-CIKs (1×10^7^ cells), CIKs (1×10^7^ cells) or PBS (control), at the area where the tumor cells had been inoculated, for five consecutive times. The subcutaneous tumor volume was measured using a caliper and estimated as follows: tumor volume (mm^3^) = 0.5 × length × width^2^.

### Statistical analysis

Values are expressed as the mean ± standard error of the mean. Statistical analysis was performed using Student’s t-test or one-way analysis of variance. Statistical analyses were performed using SPSS 16.0 software (SPSS, Inc., Chicago, IL, USA). P≤0.05 was considered to indicate a statistically significant difference.

## Results

### Transduction of DCs with a eukaryotic expression vector

**Figure 1:**
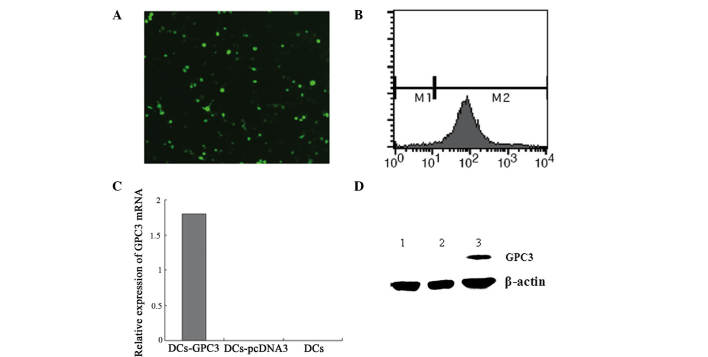
Transduction efficiency in transduced DCs. (A) DCs-GPC3 observed using an inverted fluorescence microscope (magnification, ×100). (B) Transduction efficiency in DCs. Green fluorescent protein expression was evaluated using flow cytometry 24 h after gene transduction. (C) Reverse transcription-polymerase chain reaction analysis confirmed the mRNA expression of GPC3, using DCs-pcDNA3 and DCs as the controls. (D) Western blot analysis confirmed the protein expression of GPC3. Lanes: 1, DCs-pcDNA3; 2, DCs; 3: DCs-GPC3. DCs, dendritic cells; GPC3, glypican 3.

DCs were transduced with pGFP-GPC3 to analyze the transduction efficiency. Positive GFP expression was detected in ~51% DCs, as determined using fluorescence microscopy (Fig. 1A) and flow cytometry (Fig. 1B). At 48 h after transduction, RT-PCR and western blot assays were performed to detect the expression of GPC3 in DCs. GPC3 was specifically detected in the DCs-GPC3, but not in the DCs-pcDNA3 or DCs (Fig. 1C and D).

### Phenotypic characteristics of DCs

**Figure 2:**
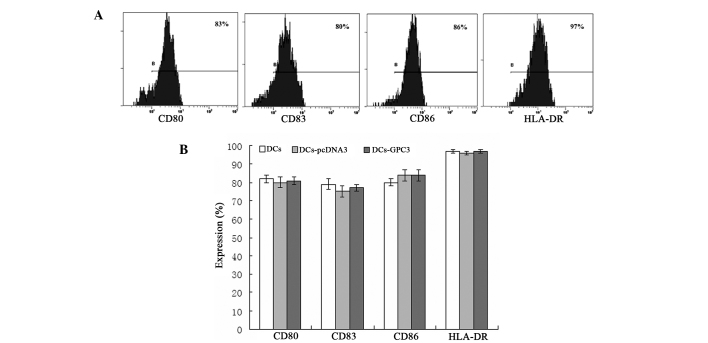
Phenotypic analysis of cultured mature DCs measured by flow cytometry. Cell surface markers were detected in mature DCs, DCs transduced with either empty vector or GPC3. (A) Expression levels of CD80, CD83, CD86 and HLA-DR, detected by flow cytometry. (B) No statistically significant differences were observed in the surface phenotypic expression of mature DCs, DCs-pcDNA3 and DCs-GPC3. Data are expressed as the mean ± standard error of the mean from three independent experiments. DCs, dendritic cells; GPC3, glypican 3; CD, cluster of differentiation; HLA, human leukocyte antigen.

The phenotypes of transduced and non-transduced mature DCs were analyzed using flow cytometry and used to detect whether transduction may affect DC differentiation and maturation *in vitro*. The results revealed that all transduced DCs exhibited a complete mature DC phenotype following stimulation with TNF-α. No statistically significant differences were observed in the expression levels of CD80, CD83, CD86 and HLA-DR between the mature DCs and transduced DCs (P>0.05; Fig. 2), indicating that gene transduction did not alter the DC surface phenotypes.

### Phenotypic characteristics of CIKs

**Figure 3:**
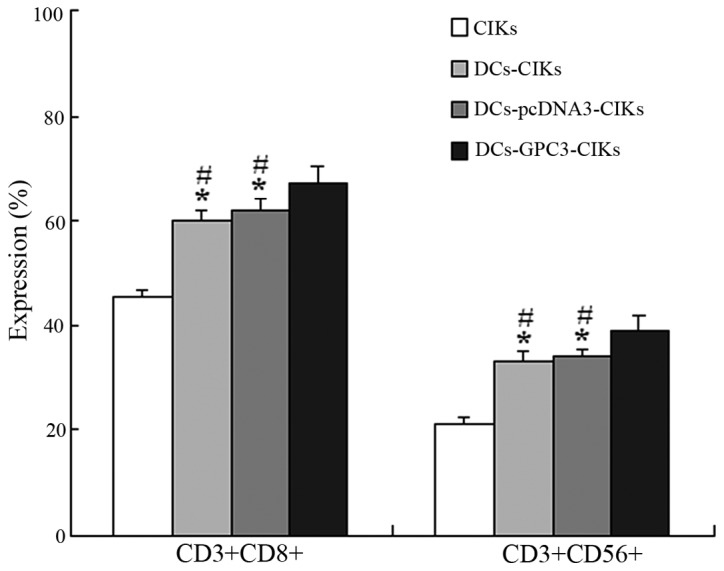
Flow cytometric phenotypic analysis of CIKs 14 days after culturing. Treatment with CIKs co-cultured with DCs or DCs-pcDNA3 significantly increased the rate of CD3^+^CD8^+^ and CD3^+^CD56^+^ compared with the autologous CIKs alone (^*^P<0.01, vs. CIKs). Furthermore, treatment with CIKs co-cultured with DCs-GPC3 significantly increased the rate of CD3^+^CD8^+^ and CD3^+^CD56^+^ compared with DCs-CIKs and DCs-pcDNA3-CIKs (^#^P<0.05, vs. DCs-GPC3-CIKs). Data are expressed as the mean ± standard error of the mean from three independent experiments. DCs, dendritic cells; CD, cluster of differentiation; CIKs, cytokine-induced killer cells.

The proportion of CD3^+^CD8^+^ and CD3^+^CD56^+^ cells was found to be significantly higher in DCs-CIKs and DCs-pcDNA3-CIKs compared with the autologous CIKs alone (P<0.01; Fig. 3). In addition, the proportion of CD3^+^CD8^+^ and CD3^+^CD56^+^ cells was significantly higher in the DCs-GPC3-CIKs compared with the DCs-CIKs and DCs-pcDNA3-CIKs (P<0.05; Fig. 3).

### Intracellular IFN-γ secretion

**Figure 4:**
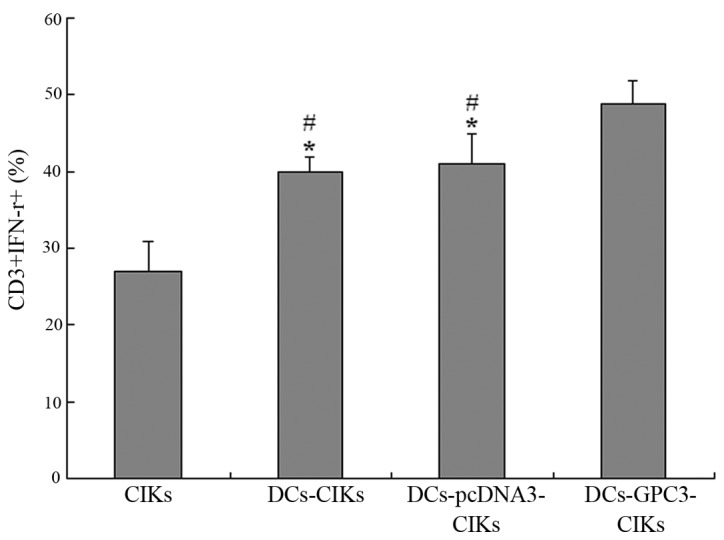
Intracellular IFN-γ-secreting CIKs determined by flow cytometry. IFN-γ secreted by DCs-pcDNA3-CIKs and DCs-CIKs was significantly higher compared with the CIKs alone (^*^P<0.01, vs. CIKs). IFN-γ secreted by DCs-GPC3-CIKs was significantly higher compared with the DCs-pcDNA3-CIKs and DCs-CIKs (^#^P<0.05, vs. DCs-GPC3-CIKs). Data are expressed as the mean ± standard error of the mean from three independent experiments. DCs, dendritic cells; CIKs, cytokine-induced killer cells; GPC3, glypican 3; IFN, interferon.

Flow cytometric analysis was used to detect the intracellular IFN-γ secretion, representing specific activation, by autologous CIKs, CIKs co-cultured with DCs that were transduced with an empty vector or GPC3. When CIKs were co-cultured with DCs-GPC3, 49% CIKs were found to secret IFN-γ. By contrast, 41 and 40% CIKs secreted IFN-γ when co-cultured with DCs-pcDNA3 or DCs, respectively (P<0.01). In addition, 27% non-transduced CIKs were found to be CD3^+^ and IFN-γ^+^ (P<0.01; Fig. 4). These results indicated that DCs transduced with GPC3 and matured with TNF-α were able to process and present GPC3 protein, resulting in the effective induction of functional CIKs, indicated by IFN-γ production.

### Induction of marked specific cytotoxic activity against HCC cells

**Figure 5:**
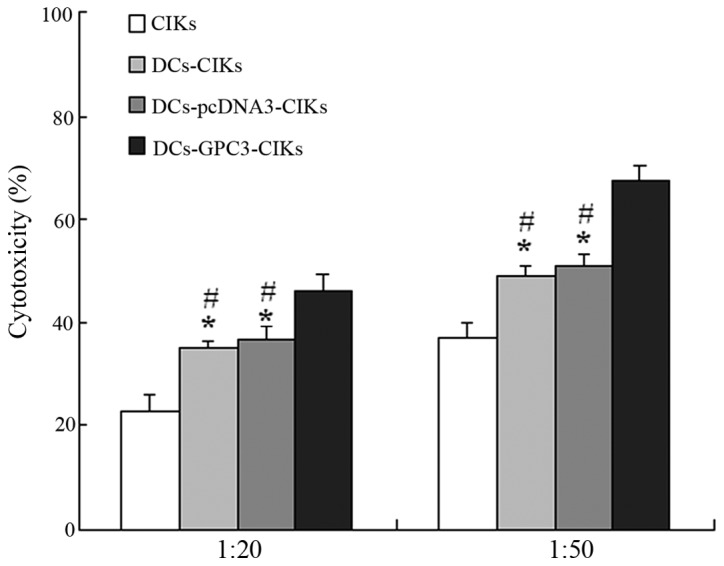
Cytotoxicity measured by a lactate dehydrogenase assay, using GPC3-expressing HepG2 cells as targets. The specific CIKs responses against HepG2 cells were investigated at effector to target ratios of 20:1 and 50:1. The cell lysis percentage in DCs-pcDNA3-CIKs and DCs-CIKs was higher compared with CIKs alone (^*^P<0.01, vs. CIKs). DCs-GPC3-CIKs presented significantly greater lysis capacity against HepG2 cells, when compared with DCs-pcDNA3-CIKs and DCs-CIKs (^#^P<0.01, vs. DCs-GPC3-CIKs). Data are expressed as the mean ± standard error of the mean from three independent experiments. DCs, dendritic cells; CIKs, cytokine-induced killer cells; GPC3, glypican 3.

In the LDH cytotoxic analysis, HepG2 cells were used as the target cells at various E/T ratios (20:1 and 50:1) to evaluate the specific cytotoxic activity. The results demonstrated that the cytotoxic activity against GPC3-expressing HepG2 cells was considerably increased in DCs-GPC3-CIKs compared with the other effector cells at the two E/T ratios (P<0.01; Fig. 5). Furthermore, the cytotoxic activity in DCs-pcDNA3-CIKs and DCs-CIKs was higher when compared with the CIKs alone (P<0.01; Fig. 5).

### Inhibitory effects on HepG2 cell-induced tumor growth in vivo

**Figure 6:**
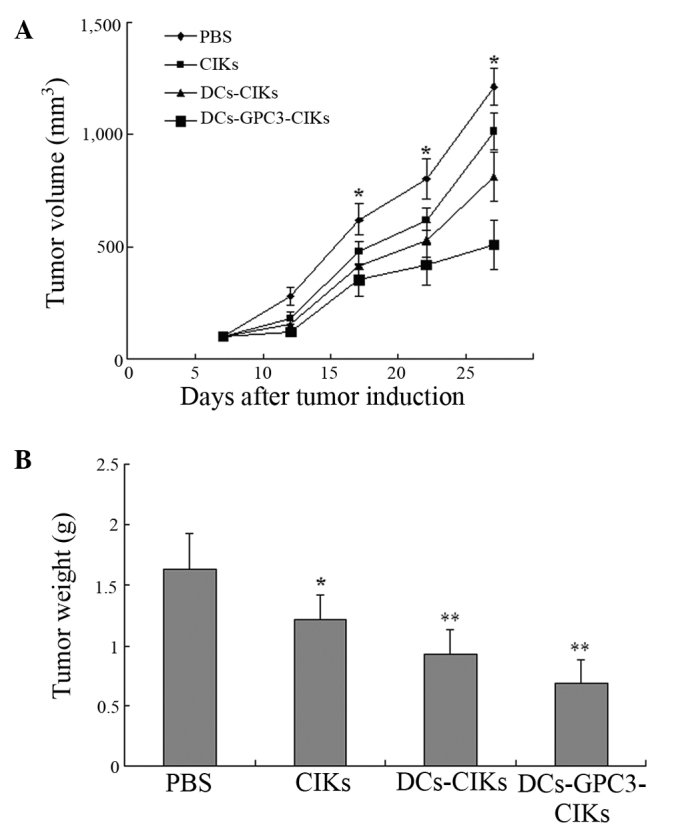
Inhibitory effects of effector cells was observed on HepG2 cell-induced tumor growth in tumor-bearing nude mice. (A) Tumor volume and (B) tumor weight in the DCs-GPC3-CIKs were found to be the smallest, followed by DCs-CIKs, CIKs and PBS. Data are expressed as the mean ± standard error of the mean from three independent experiments. ^*^P<0.05 and ^**^P<0.01, vs. PBS group. DCs, dendritic cells; CIKs, cytokine-induced killer cells; GPC3, glypican 3; PBS, phosphate-buffered saline.

The inhibitory effects of each effector cell on HepG2 cell-induced tumor growth in tumor-bearing nude mice are shown in Fig. 6. The tumor volume and weight were found to be the highest in the DCs-GPC3-CIKs group, followed by the DCs-CIKs, CIKs and PBS control groups. These results indicated that DCs-GPC3-CIKs were the most effective in inhibiting the growth of HepG2 cells *in vivo*.

## Discussion

Personalized adoptive immunotherapy may allow for more precise and optimal treatment, in order to lower the recurrence and metastasis rates of malignant tumors (24). In the present study, an immunotherapy was developed, aiming to target GPC3-expressing HCC cells for the treatment of HCC. The present study identified several key points in the development of personalized adoptive immunotherapy for HCC. An interaction was detected between DCs transduced with the GPC3 gene and CIKs, resulting in augmentation of special cytotoxicity of CIK subsets against GPC3-expressing HCC cells *in vitro*. In addition, the results revealed that gene nucleofection may be a promising approach for TAA loading. Furthermore, HepG2-induced tumor growth *in vivo* was found to be effectively inhibited by DCs-GPC3-CIKs. Finally, the marked inhibitory potential of DCs-GPC3-CIKs on HepG2-induced tumor growth may be associated with antitumor cytokines, such as IFN-γ.

Identifying reliable biomarkers is essential in order to personalize the cancer treatment of each patient through a baseline assessment of tumor gene expression and/or immune profile to optimize the therapy for the highest therapeutic success possibility (25). Several features of the expression of GPC3 on the surface of HCC cells indicate that novel immunotherapeutic approaches for HCC may be generated by targeting the GPC3 protein. Firstly, GPC3 is a membrane protein overexpressed in 70–90% of HCC cases and five HCC previously investigated cells lines (HepG2, Huh7, Hep3B, MHCC97-H and SMMC-7721) (25,26). In addition, GPC3 is not expressed in normal and cirrhotic liver tissues or in benign hepatic lesions. A previous functional analysis revealed that GPC3 promotes HCC cell migration and invasion, which may lead to tumor progression (27). Finally, clinicopathological studies have indicated that GPC3 expression correlates with poorly-differentiated HCC tumors with intrahepatic metastasis, which is a leading cause of post-surgical recurrence and reduced patient survival rates (16,29). The feasibility of GPC3 targeting for antibody or DC-based immunotherapy has been investigated in a number of studies (16,30–32).

The induction of a specific GPC3 immune response is a crucial factor for the design of immunotherapeutic strategies against cancer. Application of DC-based immunotherapy strategies is promising; however, enhancing the immunogenicity of DCs is essential. In addition, CIKs that exhibit nonspecific cytotoxicity against tumor targets but lack antitumor specificity are required. The co-cultivation of TAA-loaded DCs with autologous CIKs generates effective antitumorigenic cells (DCs-TAA-CIKs) and appears to compensate for their individual deficiencies and enhance their marked and specific antitumor immune effects (33). In the present study, nucleofector technology was selected, as previous studies have identified that nucleofection is an efficient and safe nonviral method of gene transfer into DCs without affecting the pivotal properties of DCs, which then may be used as cellular vehicles for the delivery of TAA (34,35). Despite the efficient transduction and high GPC3 expression achieved in DCs, the results of the present study revealed that all transduced DCs exhibited a complete mature DC phenotype following stimulation with TNF-α, which demonstrated that the nucleofector and gene expression did not affect the DC’s maturation and functional change toward effective presentation of specific antigens.

In order to develop an effective cancer therapy, at least three important aspects must be achieved. These aspects include obtaining sufficient effector cells, producing effector cells with a high cytotoxic activity against tumor cells and generating effector cells with specific cytotoxicity for the target cell. In the present study, an *in vitro* experiment was used to compare the autologous CIKs, DCs-CIKs, DCs-pcDNA3-CIKs and DCs-GPC3-CIKs. Co-culturing with autologous DCs-CIKs was found to enhance the cytotoxic activity against HepG2 cells compared with CIKs alone. In addition, DCs-GPC3-CIKs induced the highest cell death activity on HepG2 cells compared with other effector cells. This increment in the CIK specific cytotoxic activity when co-cultured with DCs transduced with the GPC3 gene is possibly due to an increment in the proportion of CD3^+^CD8^+^ and CD3^+^CD56^+^ cells plus a larger number of cells actively differentiating and proliferating. Another feature of CIKs is the production of effector cytokines, including IFN-γ, which enable the effector cells to potentially tilt the immune response toward the type I T helper cell and type I CD8^+^ T cell direction (NEW 3 - 36). In addition, the increased secretion of IFN-γ may further irritate autocrine CIK subsets (37). The present results demonstrated that CIKs were strongly stimulated by DCs-GPC3 with high levels of IFN-γ production, suggesting the presence of a possible mechanism of cytotoxicity. These results are in accordance with previously reported data (15,38), which demonstrated that GPC3 mRNA transfected DCs generated functional GPC3-reactive T cells, as revealed by IFN-γ production and effective lysis of GPC3-expressing HCC cells. With a substantial increase in cytotoxicity on a per cell basis and a higher proliferative response, CIKs presented a >70-fold increase in total cytolytic activity per culture when compared with other T-lymphocytes generated from peripheral blood, including lymphokine-activated killer cells and NK cells (13). The results of the current study indicated that co-cultivation of GPC3-loaded DCs with autologous CIKs may provide specific anti-HCC effective cells.

A previous study demonstrated that inhibition of the GPC3 expression of HCC cells through RNA interference reduced the tumorigenicity in nude mice, which indicated that GPC3 may be a potential molecular target in HCC therapy (28). In further *in vivo* experiments, the present study identified that tumor nodule formation in nude mice, induced by HepG2 cells, was suppressed significantly following treatment with DCs-GPC3-CIKs, indicating that specific CIKs induced by DCs-GPC3 targeting of GPC3-expressing HCC cells exhibited strong antitumor activity against HepG2 xenografts in mice.

In conclusion, the personalization of immunotherapy using DCs-GPC3-CIKs may provide an adjuvant treatment method to conventional therapeutic modalities, decreasing the recurrence rates and improving the overall survival rates of HCC patients. The precise mechanism of growth inhibition requires further examination in future studies.
